# Mass Spectrometry in Chiral Analysis and Synthesis

**DOI:** 10.1002/chir.70126

**Published:** 2026-07-31

**Authors:** Brison A. Shira, Mahdiyeh Shahi, R. Graham Cooks

**Affiliations:** ^1^ Department of Chemistry Purdue University West Lafayette Indiana USA

**Keywords:** chiral analysis, clinical analysis, high‐throughput analysis, microdroplet chemistry, molecular clusters, serine octamer

## Abstract

Mass spectrometry (MS) has a role to play in chiral chemistry, in part through methods based on microdroplets and non‐covalent molecular clusters. High‐throughput MS reaction screening and chiral anal0ysis use microdroplet sprays to perform accelerated nanoscale chemistry, including the synthesis of heterocyclics and other drug candidates using new high‐throughput automated instrumentation. The kinetic method of chiral analysis measures competitive dissociation reactions of molecular clusters (usually trinary coordination complexes) to determine enantiomeric excess (e.e.). A very different molecular cluster, the serine octamer, an active research topic for two decades, may hold implications in the search for the origins of homochirality. Lastly, the recently reported direct discrimination of enantiomers using an ion trap mass spectrometer (ITMS) is revisited. These four examples, combined with recent findings concerning chiral post‐translational modifications in cancer biology, represent exciting research developments that deploy the advantages of MS (throughput, molecular specificity, and sample size) to improve chiral synthesis and analysis.

## Graphical abstract

Chiral molecules have the same mass, yet there are MS approaches that can distinguish chiral molecules. MS‐based chiral chemistry matters in fundamental physical experiments, industrial applications, and clinical analysis.

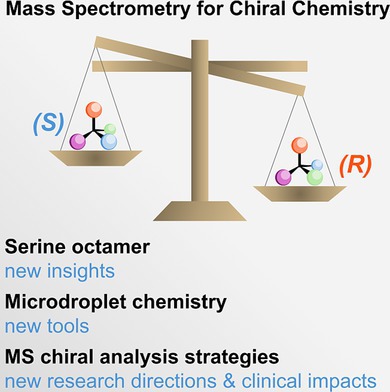

## 1 Introduction

### 1.1 What Can Mass Spectrometry (MS) Say About Chirality?

Ostensibly, the prospects of mass spectrometry (MS) measurement for chiral analysis are limited [1]. The primary measurement variable of MS is mass (through mass‐to‐charge ratio, *m/z*) and stereoisomers have identical masses. Nevertheless, there are many examples of chiral MS analysis. These are growing in importance and include the quantitation of enantiomers from mixtures [2, 3, 4], high‐throughput chiral analyses [5], experiments involving chiral recognition [6], and contributions to the understanding of chiral molecules in pathology [7, 8].

As part of the collection commemorating the Chirality 2025 conference in New York City, this article is a “snapshot” of recent progress. Our goal is to inform chirality specialists of recent developments in MS while also calling the attention of MS specialists to emerging directions in research involving chirality. Beginning with a survey of chiral MS strategies, the role of microdroplets in high‐throughput analysis and asymmetric synthesis is next discussed. Research concerning the serine octamer cluster is revisited. We conclude by reexamining the recent report of direct MS chiral differentiation [9].

## 2 Discussion

### 2.1 Strategies for Chiral MS

Although it is easy to dismiss mass as a distinguishing feature between enantiomers, the intrinsic molecular parity violation energy (MPVE) [10, 11] gives chiral molecules energies (and hence masses) that differ by approximately 10^−11^ J mol^−1^. The difference in mass follows from Einstein's relationship (Equation 1):

(1) $$E={\mathrm{mc}}^{2}$$

Such a mass difference between enantiomers implies a straightforward way to do chiral MS. Practically though, measuring the ca. 10^−25^ amu mass difference that would result from MPVE is not possible [12]. A variable besides mass is then required, and fortunately, MS instrumentation *can* measure a variety of physical properties. For chiral MS, kinetic and spectroscopic measurements are the most valuable.

Spectroscopic techniques can avoid the need for molecular complexation as light itself can supply the asymmetry required. In a typical implementation, polarized light is used to enantiospecifically detach an electron from an analyte anion [13, 14]. These experiments are elegant but require sophisticated energy sources and detection systems [15, 16]. Additionally, the measured difference between chiral analytes can be small, limiting measurement accuracy [17].

Ion mobility spectrometry (IMS), which is often coupled with MS, offers several avenues for chiral determination [18]. Its measurement variable, rotationally averaged collision cross section (CCS), is determined by colliding an ion with a buffer gas along a flight path. In certain cases, chiral biopolymers can be differentiated directly [19], because the inclusion of “wrong‐handed” moieties perturbs folding. For smaller molecules, derivatization or complexation is often used to provide chiral discrimination by CCS [20, 21, 22, 23].

Kinetic methods enjoy wide use in chemical analysis; they entail processes that exhibit rate differences sensitive to a given property, such as stereochemistry [24]. One widespread kinetic method, the “MS kinetic method,” allows chiral analysis. The procedure is as follows: (i) Spike a sample with a reference compound (Ref, e.g., phenylalanine, or a chelating ligand, often a short peptide) and a metal ion (M^II^, e.g., Cu^2+^); (ii) ionize this sample to form the cluster [Analyte + 2Ref + M^II^ − H]^+^; and (iii) mass‐select this cluster and subject it to tandem MS to measure the rates of competing dissociation pathways: the losses of Ref versus Analyte. In the coordination complex, enantiomers have unequal non‐covalent contacts, giving dissociation energies, ΔG^‡^, that depend on the chiral composition of the cluster [2]. The method's advantages are its speed, sample size, sensitivity, and matrix tolerance. Its limitations include the fact that the selection of Ref and M^II^ is determined empirically and a calibration curve is required [25]. Still, this strategy is robust and it applies to high‐throughput assays [5].

### 2.2 Accelerated Reactions in Water Microdroplets

Reaction acceleration in microdroplets has become a vast topic since its first observation in 2011 [26]. Recently, a consensus understanding of the acceleration mechanism has emerged, as described in several reviews [27, 28, 29]. (Debate persists as to the initiating steps. Whereas some have invoked species such as HO^●^ and H_2_O^+●^/H_2_O^─●^ as reaction initiators [29, 30, 31], others remain skeptical of the role played by such species [32, 33, 34, 35].) Accepted reasons for acceleration are partial solvation, strong interfacial electric fields, and advantageous mass transfer. Two areas of microdroplet chemistry deserve discussion in the context of stereochemistry: droplet‐enabled HT‐MS analysis and the prospects of using microdroplets for green asymmetric synthesis.

#### 2.2.1 Analysis

Microdroplet chemistry is intertwined with MS for historical and practical reasons. Early work with microdroplets used MS [26, 36], and this pairing allowed online execution of a reaction and the analysis of its products within milliseconds. Purdue's high‐throughput mass spectrometry (HT‐MS) user facility is a mature realization of this concept. The system offers bioassays, accelerated organic synthesis, and product collection.

Microdroplet phenomena enable HT‐MS when desorption electrospray ionization (DESI) is used to generate microdroplets. DESI is unique because it is array compatible and it is suited to chiral chemistry because it forms the molecular clusters needed for the kinetic method [37]. The array format allows use of a robotic fluid handler to prepare the combinations of chiral references and metal ions needed for method development and for the creation of calibration curves. Automation and the intrinsic speed of MS make chiral MS quantitation by the kinetic method faster than other common methods by up to two orders of magnitude [5].

Of particular interest is the chiral oncometabolite 2‐hydroxyglutarate (2HG) [7, 8]. Deploying chiral HT‐MS for this clinical analysis is a priority because 2HG has diagnostic value as a marker for cancer [38]. Accumulation of D‐2HG is frequently associated with cancer. It is a phenotype of isocitrate dehydrogenase (IDH)–mutant tumors, the most frequently mutated metabolic enzyme in cancer [39], but there are also scenarios in which excess L‐enantiomer is diagnostically indicative [40]. Chiral determination of 2HG is therefore important in clinical diagnosis and pathology, a fact made all the more apparent with recent work showing that 2HG drives posttranslational modifications with stereochemical preference [8]. The kinetic method can be used to determine 2HG chirality even in brain cancer biopsies—an analysis for which it is highly advantaged, owing to its small sample sizes.

#### 2.2.2 Synthesis

Microdroplets are known to allow both chemical and nanomaterial syntheses [41, 42, 43]. They do not require catalysts and use non‐problematic solvents, and so are emerging as a means to green synthesis, including for multicomponent chemical transformations [44, 45]. Continued progress includes the development of specialty devices designed for microdroplet synthesis at scale. Acceleration of reactions in microdroplets allows small‐scale molecular synthesis during droplet flight (millisecond timescale) with a variety of transformations being performed and with product collection for bioassay [43, 46, 47].

**FIGURE 1:**
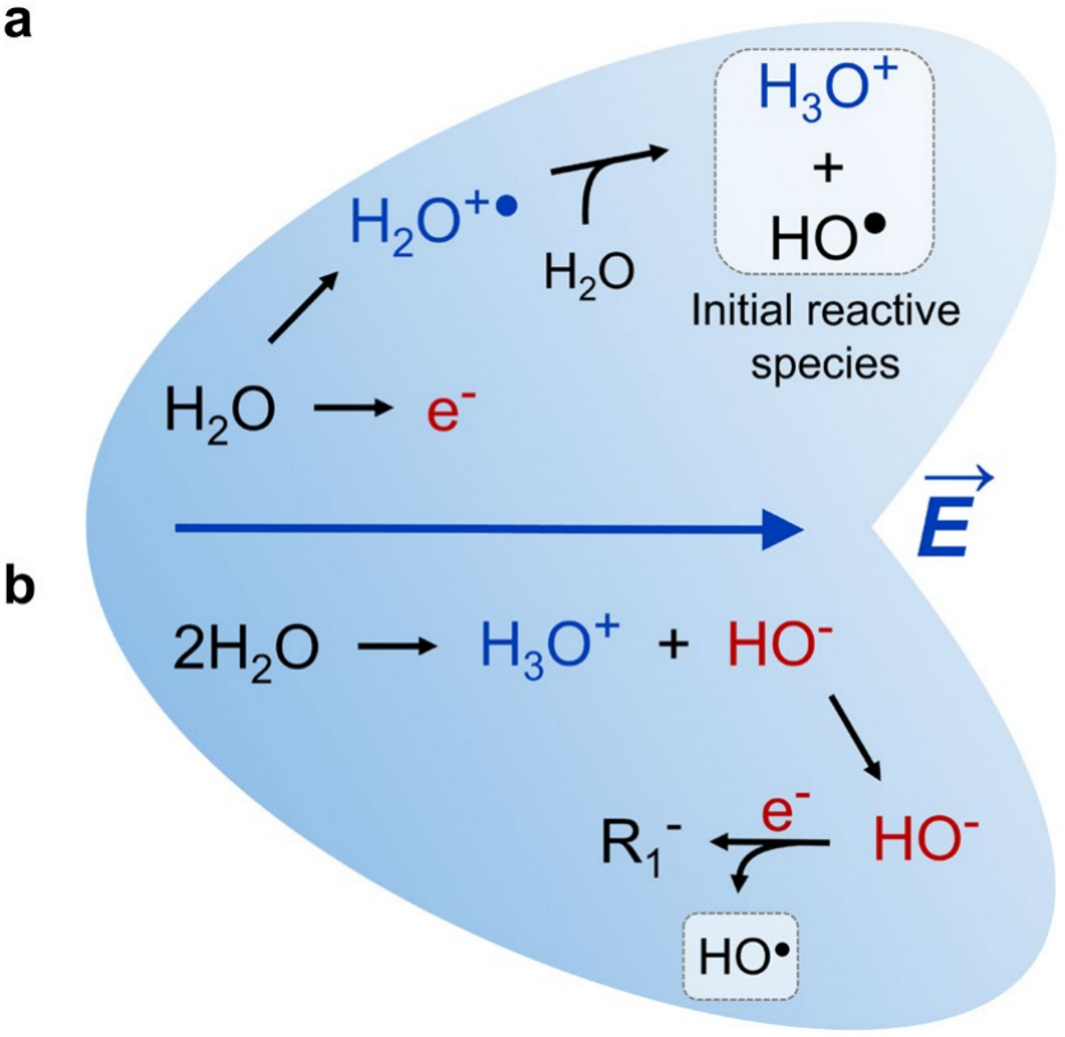
Graphical summary of reaction initiation at the microdroplet air/water interface. (a) Field ionization at the intrinsic interfacial electric field, $\vec{E}$, forms the reaction initiating species (ref. [29]). (b) The same process initiated by electron transfer from OH^─^, as suggested in ref. [30].

Interfacial phenomena are central to microdroplet reactions (e.g., surface affinity mechanisms [29] and the electric field of the air/water interface [48, 49, 50]). One consequence of the electric field is that field ionization, a barrierless process in which electrons quantum tunnel from the droplet surface, can generate strong redox species responsible for aspects of microdroplet reactivity [27, 51]. Recently, Min's group has added a detailed Marcus‐theory analysis in which OH^─^ similarly engages in a barrierless electron transfer at the droplet interface (Figure 1) [30, 31].

Aside from initiating microdroplet reactions, the strong electric field also implies the possibility of aligning reagent molecules at the interface, which may be the first step toward green asymmetric synthesis. It is known that interfacial assembly [52, 53] allows enantioselective reactions [54]. If molecular clusters (such as the serine octamer) [55] or sterically constrained prochiral molecules accumulate at the interface, microdroplets may provide an accessible form of confinement for asymmetric synthesis.

### 2.3 Serine Octamer Molecular Clusters

The serine octamer (Ser_8_) has galvanized the MS community like few other species. First observed in 2001, this molecular cluster illustrates the practical utility of chiral MS and has potentially important implications for homochirogenesis [56].

**FIGURE 2:**
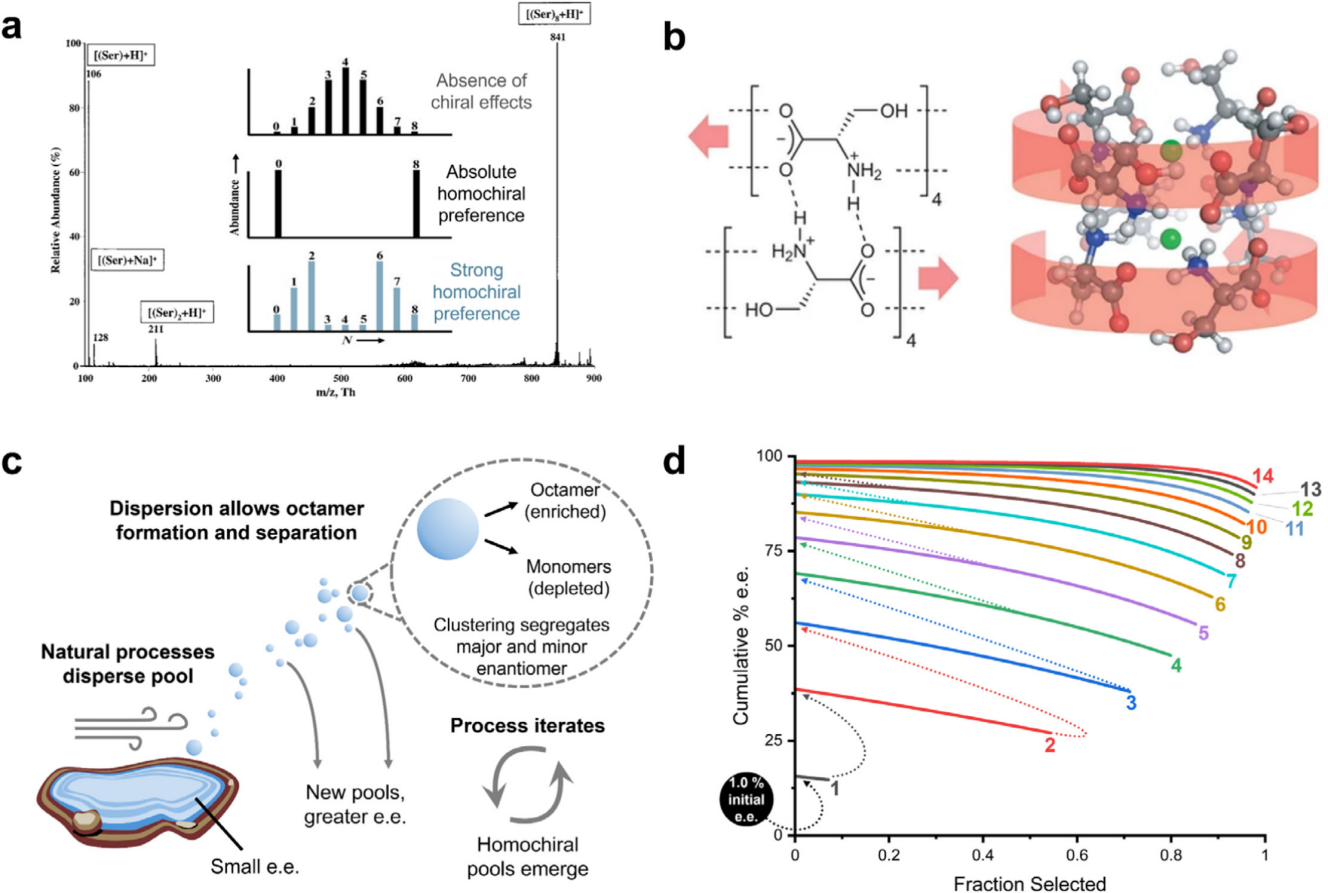
(a) Ser_8_H^+^ is a strongly magic cluster observed in MS experiments; inset bar graph shows empirical chiral preference obtained from racemic monomer pool (reprinted with permission from Cooks et al. [61]. Copyright 2001 American Chemical Society). (b) Combined theory and spectroscopic experiments indicate that Ser_8_Cl_2_^2─^ has a symmetric structure, suggesting that the neutral cluster may also be highly symmetric (reproduced with permission from Springer Nature [62]). (c) Flow diagram depicting how pools of non‐racemic serine could give non‐zero e.e. through octamer clustering. (d) Results of a simple numerical simulation in which Ser_8_ is allowed to form, sampling a large pool with small e.e. to form a small pool with larger e.e.; this process iterates 14 times to achieve high enantiomeric excess (see also Figure S1).

Ser_8_ is usually observed as a gas‐phase cluster ion in MS experiments. Its special properties are its magic number and strong chiral preference. Ser_8_ is a textbook example of a magic number cluster, meaning its formation is much more likely than other serine clusters (e.g., Ser_9_ or Ser_7_) [57, 58, 59, 60]. Strong chiral preference—the tendency of the cluster to form from all L‐ or all D‐monomers—is observed in Ser_8_ formation (Figure 2a) [61]. This preference extends to substitution reactions in which amino acids, sugars, etc. are incorporated into the octamer enantioselectively [62].

There are other examples of chiral molecular clusters [63, 64, 65, 66, 67, 68]. Worth mentioning are the host/guest complexation reactions [69, 70] and the use of fast atom bombardment to extract these intact complexes from solution [71, 72]. MS is advantaged in these types of experiments because it can directly isolate the clusters in question and probe their non‐covalent attractions. Despite being experimentally limited to gas‐phase species, clustering interactions observed with MS produce data relevant to chemical processes of much broader importance, such as fibril formation [73, 74].

The chiral properties of a molecular cluster are largely attributable to non‐covalent contacts. In the case of Ser_8_ where magic number behavior is also involved [75], high symmetry is also usually present. Spectroscopic evidence suggests that this is the case (Figure 2b) [76]. Ordered non‐covalent contacts between chiral monomers provide a plausible explanation for its chiral preference [77, 78, 79, 80]. Yet, protonation to make Ser_8_H^+^ distorts the cluster's structure, eliminating its symmetry [81]. This raises the question of whether the neutral octamer exhibits even greater enantioselectivity and whether other neutral clusters might also be strongly chiral.

Accordingly, it is worth considering octamer formation as a vehicle for chiral resolution. Ser_8_ allows an aliquot of a solid or aerosolized sample to enter the gas phase while enacting a strong bias toward homochirality. Next, it may be blown away from its initial pool (Figure 2c,d and Figure S1). One significant point is that even for a racemic sample, Ser_8_ forms clusters that tend not to contain equal amounts of each enantiomer, meaning that each individual cluster has an e.e. and can seed new pools that are now non‐racemic, amplifying any fluctuation away from 0% e.e [82, 83]. Furthermore, as the preferential sampling of Ser_8_ depletes the e.e. of an enriched pool it is possible for other processes, especially thermal racemization, to return the residual pool to its starting e.e., where additional cycles may occur. Re‐racemization is key to this model because without it, the amplified enantiomer would be depleted and any resolving effect null.

### 2.4 Direct Determination of Molecular Chirality With MS

More than 2 years have passed since the publication of a novel method of chiral analysis based on direct determinations with an ion trap mass spectrometer (ITMS) [9]. Reception in the literature has been muted [23, 84, 85, 86]. Nevertheless, their results are important because a general, rapid, sensitive, and matrix tolerant chiral measurement would be a major advance [87]. Understanding and deploying this measurement therefore represents an important challenge for the field of chiral MS.

**FIGURE 3:**
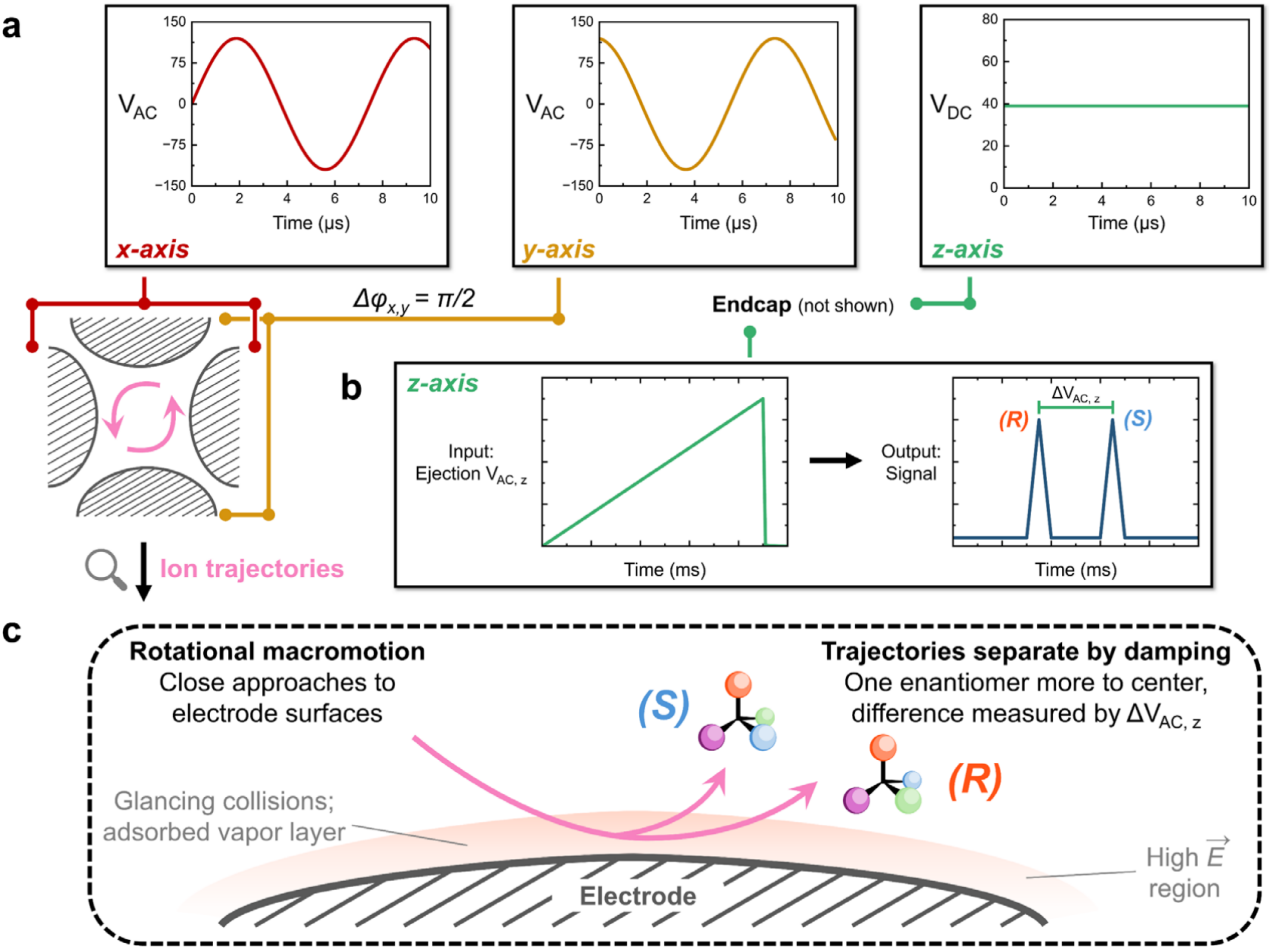
Graphical summary of the chiral ITMS demonstration. (a) Resonant supplemental waveforms (π/2 out‐of‐phase) are applied to induce circular trajectories that separate enantiomers. (b) Separation is measured by ramping ejection amplitude, V_AC_. (c) Our interpretation. Close approach to electrodes sends ions into the region of extremely high electric fields and collisions with adsorbed gas, causing measurable trajectory differences.

This experiment's data are compelling. As usual in MS, the analytes are gas‐phase ions that are differentiated by their response to electric fields. Here, *m/z* is not the focus; instead, alternating current waveforms were applied to the electrodes of an ITMS, causing excitation that differently affects the trajectories of enantiomeric analyte ions. Separation occurs in the amplitude (V_AC_) required to eject the enantiomers. As in chromatographic analysis, the strength of retention in an asymmetric environment differentiates the enantiomers. Although chromatography relies on Van der Waals attractions and sterics, in the trap, electric fields and their influence on ion/neutral collisions cause separation in the V_AC_ required to eject each enantiomer. To explain their findings, the authors added a molecular propellor effect argument to the ion cloud profiling theory they previously developed [88]. We build on this (Figure 3).

While the ion cloud model accounts for previous demonstrations of isomer separation because ions with different CCS experience different motional damping during their residence in the ITMS [89], it does not explain how enantiomers are separated. Enantiomers have identical *m/z* and CCS, so measurable differences are not expected. The authors invoke the macromotion of the ions in the trap as a symmetry‐alleviating condition that causes differences in motional damping and thus ion cloud profile. To achieve this, a circular ion trajectory is induced with a supplemental excitation waveform, which is thought to engage a molecular propeller effect (a hydrodynamic argument) [90]. This, the authors explain, accounts for the differences in the ion cloud profile measured by the V_AC_ scan.

The propellor effect is established in the literature, but only for solution‐phase chiral separation [90, 91], where the medium through which a molecule passes contains many molecules compared to the ITMS at 10^−5^ Torr. This hypothesis does not fully explain the demonstration because this effect would require (i) a strong propensity for the chiral analyte's dipole to align with the electric field and (ii) while aligned, a continuous drag must differentiate the CCS through rotational/translational coupling. With a condensed phase propeller effect, damping forces are continuous because many solvent molecules always contact an analyte, satisfying (ii). Even so, Brownian motion interferes with the alignment of an analyte with the electric fields driving separation, complicating (i). In MS, the opposite happens, (i) alignment is likely, but (ii) continuous drag is not. The vacuum chamber is non‐continuous, and its “billiard ball” collisions between background gas and analyte ions do not produce chiral drag—otherwise all enantiomers would be separable in IMS.

As an alternative explanation, we suggest that the defocusing of ions away from the trap center and toward the electrodes by application of excitation waveforms opens the possibility of a continuous drag and the propeller effect, satisfying (ii). Indeed, vacuum chambers accumulate adsorbed gas or vapor on surfaces. This point is especially pertinent to the instruments in question that use a pulsed atmosphere/vacuum interface that periodically injects ambient air into the chamber. Layers of adsorbed gas coating the electrodes of the ITMS would explain how ions in an otherwise sparse vacuum chamber could experience many collisions with the requisite retention of molecular alignment; collisions occur in the region where the field is strongest, at the closest approach to the electrode.

When ambient air is admitted to the vacuum chamber, the material that accumulates on the walls is not a representative sample of air; the polar fraction is overrepresented, meaning that water and organic contaminants may accumulate on the electrode surface. It follows that these contaminants, which mostly originate from homochiral living organisms, could coat the electrode surface effectively, making a chiral stationary phase. Although the precise explanation is still unclear, the role of adsorbed material must be considered to explain how trapped ions might experience a sufficient density of chiral intermolecular interactions to distinguish enantiomers.

At a broader view, consider that to break the symmetry between enantiomers, asymmetric forces need to exist in three dimensions. Indeed, such conditions have been used in microwave spectroscopy [92], suggesting that chiral molecules can be directly differentiated given appropriately designed electric fields [93, 94]. In a mass spectrometer, the challenge is not the creation of an asymmetric environment, but the ability to realize asymmetric forces in a medium which chiral differences are not obfuscated by thermalization. Chiral ITMS solves this by directing ion trajectories to the extremes of the trap, where analytes experience a local environment of higher pressure but strong electric field alignment. Here, rotational/translational energy conversion could separate two ion clouds because one enantiomer more strongly interacts with the adsorbed electrode layer. There is precedent for aspects of these findings: chemical mass shift phenomena have been described in which the close approach of ions to the electrode (and regions of higher‐order fields) enables isomer and isobar differentiation [95, 96].

## 3 Conclusion

Because MS methods allow rapid experiments with very small samples, their broader adoption in chiral chemistry without associated chromatographic separation is poised to accelerate. Simply put, more experiments done more quickly with more detailed analysis will facilitate greener chiral analysis and synthesis [97]. The implications are even more profound in the application of chiral MS to clinical analysis where molecular asymmetry can indicate disease state. Chiral MS is not merely a methodological innovation but an opening for significant new measurements and insights.

## Funding

Funding was provided by Multidisciplinary University Research Initiative of the Air Force Office of Scientific Research (FA9550‐21‐1‐0170) via Stanford University (subaward 62741613‐204669), Waters Corporation, and Agilent Technologies via an ACS Analytical Chemistry Graduate Fellowship (to B.A.S).

## Supporting information


**Figure S1:** (a) Graphical representation of chiral enrichment of serine via formation and selective removal of the octamer, showing how in principle, the % enantiomeric excess of the collected serine increases with the number of Ser_8_ formation and deposition steps. (b) Result of simple model showing % e.e. of selected material after single processing through the octamer for each initial % e.e. (c) Cumulative result of 14 processing steps.

## Data Availability

Data are available from the corresponding author upon reasonable request.
