# Supplementary material for: Mass Spectrometry in Chiral Analysis and Synthesis
Source: Chirality. 2026 Jul 31;38(8):e70126. doi: 10.1002/chir.70126 (PMC13425062; doi:10.1002/chir.70126)
Supplement: Supplementary file 1 — Figure S1: (a) Graphical representation of chiral enrichment of serine via formation and selective removal of the octamer, showing how in principle, the % enantiomeric excess of the collected serine increases with the number of Ser8 formation and deposition steps. (b) Result of simple model showing % e.e. of selected material after single processing through the octamer for each initial % e.e. (c) Cumulative result of 14 processing steps. [file CHIR-38-e70126-s001.docx]

**Supporting Information for “Mass Spectrometry in Chiral Analysis and Synthesis”**

Brison A. Shira, Mahdiyeh Shahi, and R. Graham Cooks*

*Denotes corresponding author; email: cooks@purdue.edu

**Table of Contents**

Figure S1. [Description of numerical simulation of Ser_8_ enrichment 1](#_Toc231815806)

[References 2](#_Toc231815807)

Description of numerical simulation of Ser_8_ enrichment


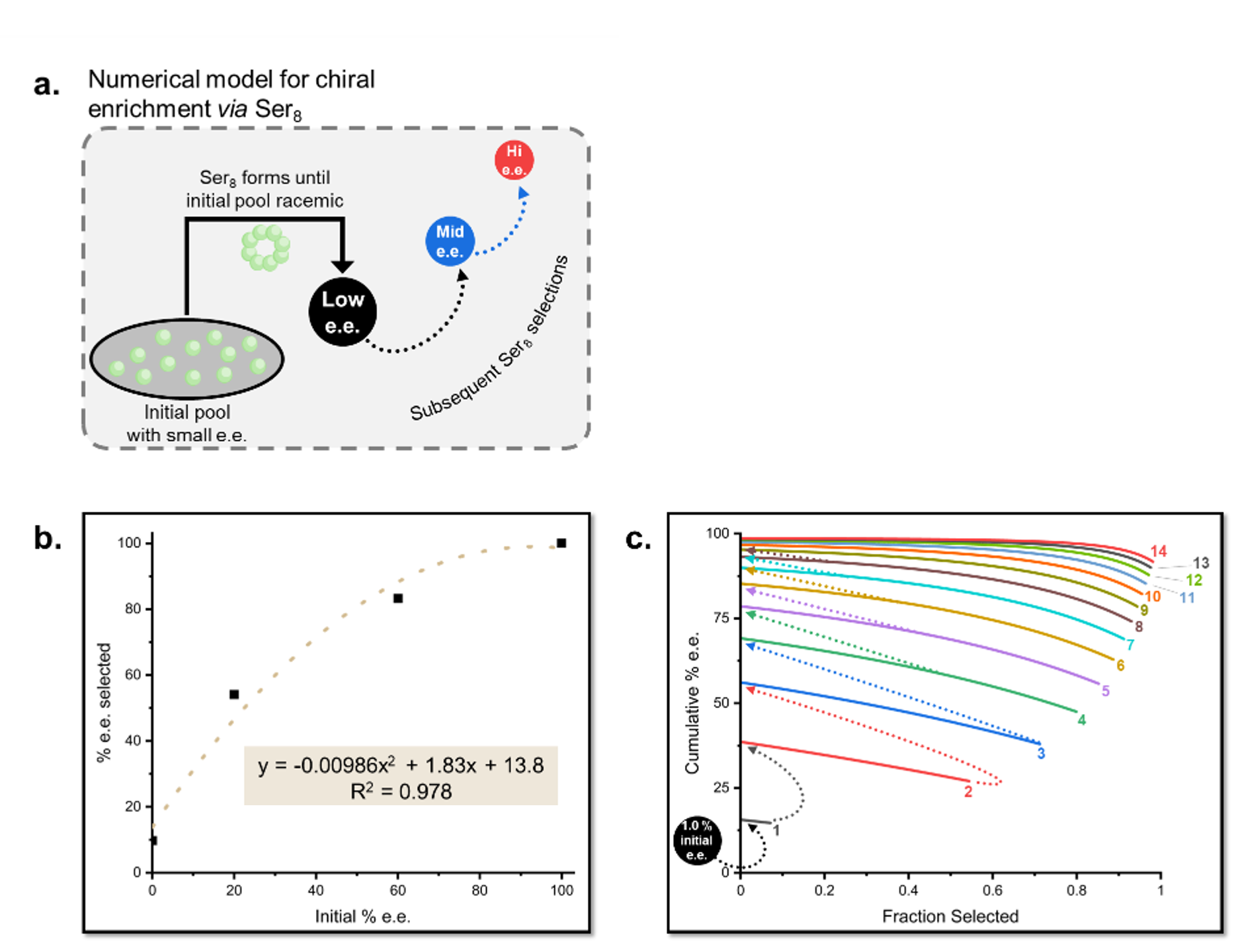


**Figure S1 a)** Graphical representation of chiral enrichment of serine via formation and selective removal of the octamer, showing how in principle, the % enantiomeric excess of the collected serine increases with the number of Ser_8_ formation and deposition steps. **b**) Result of simple model showing % e.e. of selected material after single processing through the octamer for each initial % e.e. **c**) Cumulative result of 14 processing steps.

The data plotted in main text **Figure 3d** (reproduced above in **Panel** **c** of Fig. S1 for convenience) were obtained using a simple numerical model as described below. As shown in **Panel a**, a pool of serine with a specified enantiomeric excess (e.e.) was considered. In steps of one-thousandth of the total, material was allowed to form Ser_8_ and removed from the initial pool. To determine the chiral composition of the one-thousandth of the initial pool that formed Ser_8_ clusters, data from a past study^1^ were consulted in which a sample with a known e.e. was prepared and the e.e. of the resulting octamers was determined. A quadratic curve was fit to these data to approximate the relationship between starting sample e.e. and the e.e. of the observed octamers, viz. the chiral preference of the octamer (**Panel b**). Using this relationship, the e.e. of each Ser_8_ formation event could be estimated as a function of the e.e. of the initial pool.

To simulate how e.e. could be amplified and transferred to another smaller pool, we modeled the formation of Ser_8_ from an initial pool until the original sample returned to 0% e.e. All the Ser_8_ which was formed was assumed to collect into a new pool which now had a greater enantiomeric excess than the initial pool, albeit with a smaller total size. This process was cycled, modeling how the chiral preference of Ser_8_ can give rise to progressively greater e.e., as depicted in **Panel c**, which shows how 14 cycles of octamer formation results in the formation of pools with high e.e.

References

(1) Nanita, S. C.; Takats, Z.; Cooks, R. G.; Myung, S.; Clemmer, D. E. Chiral enrichment of serine via formation, dissociation, and soft-landing of octameric cluster ions. *Journal of the American Society for Mass Spectrometry* **2004**, *15* (9), 1360-1365. DOI: 10.1016/j.jasms.2004.06.010.
